# Low baseline IFN-γ response could predict hospitalization in COVID-19 patients

**DOI:** 10.3389/fimmu.2022.953502

**Published:** 2022-09-26

**Authors:** Marion Cremoni, Jonathan Allouche, Daisy Graça, Kevin Zorzi, Céline Fernandez, Maxime Teisseyre, Sylvia Benzaken, Caroline Ruetsch-Chelli, Vincent L. M. Esnault, Jean Dellamonica, Michel Carles, Jérôme Barrière, Michel Ticchioni, Vesna Brglez, Barbara Seitz-Polski

**Affiliations:** ^1^ Clinical Research Unit Côte d’Azur (UR2CA), University Côte d’Azur, Nice, France; ^2^ Immunology Laboratory, Archet 1 Hospital, Nice University Hospital, Nice, France; ^3^ Department of Public Health, Archet 1 Hospital, Nice University Hospital, Nice, France; ^4^ Intensive Care Medicine Department, Archet 1 Hospital, Nice University Hospital, Nice, France; ^5^ Medical ICU, Archet 1 Hospital, Nice University Hospital, Nice, France; ^6^ Infectious Diseases Department, Archet 1 Hospital, Nice University Hospital, Nice, France; ^7^ Department of Medical Oncology, Polyclinique Saint-Jean, Cagnes-sur-mer, France

**Keywords:** SARS-CoV-2, viral infection, IFN-γ, functional immunoassay, predictive biomarker, personalized medicine

## Abstract

The SARS-CoV-2 infection has spread rapidly around the world causing millions of deaths. Several treatments can reduce mortality and hospitalization. However, their efficacy depends on the choice of the molecule and the precise timing of its administration to ensure viral clearance and avoid a deleterious inflammatory response. Here, we investigated IFN-γ, assessed by a functional immunoassay, as a predictive biomarker for the risk of hospitalization at an early stage of infection or within one month prior to infection. Individuals with IFN-γ levels below 15 IU/mL were 6.57-times more likely to be hospitalized than those with higher values (p<0.001). As confirmed by multivariable analysis, low IFN-γ levels, age >65 years, and no vaccination were independently associated with hospitalization. In addition, we found a significant inverse correlation between low IFN-γ response and high level of IL-6 in plasma (Spearman’s rho=-0.38, p=0.003). Early analysis of the IFN-γ response in a contact or recently infected subject with SARS-CoV-2 could predict hospitalization and thus help the clinician to choose the appropriate treatment avoiding severe forms of infection and hospitalization.

## Introduction

The severe acute respiratory syndrome coronavirus 2 (SARS-CoV-2) infection that emerged in China in late 2019 has spread rapidly around the world, causing millions of deaths, overwhelming public health services, and resulting in severe economic and social crisis. Individuals who are male, older than 60 years, and have comorbidities are at higher risk for severe COVID-19, requiring hospitalization and more frequently presenting with complications such as multivisceral failure or death (1–4). The implementation of preventive measures such as lockdown, social distancing (5, 6) and vaccination (7–9) have limited its spread. Although no curative treatment has unequivocally demonstrated its effectiveness (10), the administration of antiviral drugs in the early phase of the disease appears to decrease viral replication and pathogenesis (11–13), interferon (IFN) therapy reduces the duration and severity of symptoms, as well as mortality, if administered early (14–20), and the treatment with monoclonal antibodies results in fewer hospitalizations and deaths in immunocompromised patients (21–23).

The immune response to a viral agent, including SARS-CoV-2, involves both the innate and adaptative response. Innate immunity induced by Toll-like receptors 3 (TLR3) and TLR7/8 signaling activates effector cells to mediate viral clearance, induces inflammation through secretion of proinflammatory cytokines (e.g., IL-6 and IL-1β), produces antiviral cytokines and stimulates the adaptative immune response by activating antigen-specific T cells. Type I and II IFN (i.e., IFN-α/β and IFN-γ, respectively) are the first-line cytokines against viral infections. While many studies have focused on type I and III IFN alteration in severe forms of COVID-19 (24–34), less research has been conducted on type II IFN deficiency (35–38). However, if type I IFN is a component of innate immunity, type II IFN is involved in both innate and adaptative immune responses. Indeed, IFN-γ is produced by natural killer cells and macrophages, effector cells in innate immunity, as well as by CD4^+^ T cells of the Th1 type and CD8^+^ T cells that participate in the adaptative response.

Moreover, rapidly after the beginning of the pandemic, many authors highlighted that an excessive pro-inflammatory innate immune response could be deleterious in the defense against the virus, leading to a cytokine storm responsible for acute respiratory distress syndrome, multivisceral failure and even death. Interleukin-6 (IL-6) is one of the main cytokines involved in the cytokine storm (35, 39–43). This observation made anti-IL-6 and corticosteroids first-line treatments for severe COVID-19. However, the evidence of excessive secretion of pro-inflammatory cytokines, including IL-6, is often late in the infection and does not allow the implementation of preventive measures. It is likely that a deficiency in interferons, responsible for a persistence of the virus by defect of clearance, can support the overexpression of pro-inflammatory cytokines at the origin of the cytokine storm (35, 41).

To our knowledge, no study has investigated the IFN response at an early stage of SARS-CoV-2 infection, or even before contamination, to detect preexistent immune dysfunction in subjects who may subsequently progress to a severe form of the disease. Thus, we hypothesize that dysregulation of the basal IFN-γ response, as assessed by an easy to perform functional immunoassay, promotes severe forms of COVID-19 requiring hospitalization. A clinically applicable blood biomarker identifying patients with dysregulated IFN-γ response could optimize management by directing the prescription of antivirals and/or IFN and/or monoclonal antibodies to patients likely to benefit from them, thus reducing the number of hospitalizations, while avoiding deleterious over-prescription and potential associated adverse effects in those for whom the treatment would not be of interest.

## Materials and methods

### Participants, data collection and ethics statement

We performed a prospective monocentric longitudinal and ancillary study at the Nice University Hospital, France. The participants included were extracted from three prospective monocentric cohorts: (i) patients recruited during an infectious diseases or emergency room consultation following COVID-19 symptoms, or a contact case between March 2020 and January 2022 (CovImmune 1 study, NCT04355351); (ii) patients recruited by partner laboratories during a positive RT-PCR for SARS-CoV-2, performed in the context of suggestive symptomatology, contact case, or health pass, between August 2021 and November 2021 (CovImmune 1 study, NCT04355351); (iii) voluntary participants from the general population monitored systematically and periodically since July 2020 as part of an epidemiological study in the context of COVID-19 (CovImmune 2 study, NCT04429594). COVID-19 positive participants for whom we had a stimulated blood sample either a) within one month prior to infection or b) within five days after the first symptoms of the disease or after a close contact with a COVID-19 case, were enrolled in this study. Demographic, clinical, biological, and outcome data were collected by the study investigators and then centralized in an anonymized database. Written informed consent was obtained from all study participants.

### Blood collection and immunoassays

**Figure 1:**
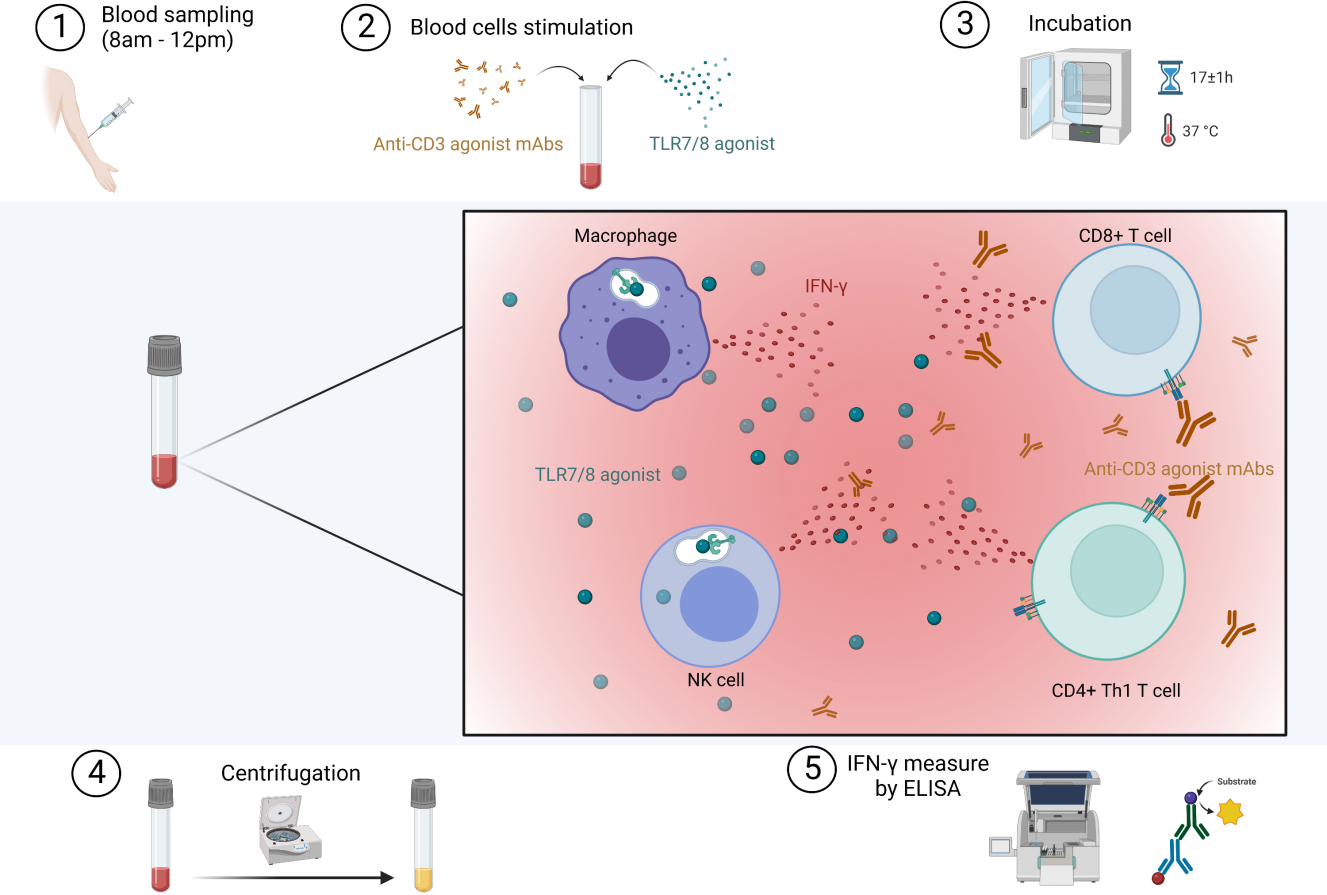
Procedure for the *in vitro* stimulation of immune cells in whole blood. Immune ligands mimic pathogen-associated molecular patterns that activate innate immune cells through TLR7/8 and T cells through CD3. IFN, interferon; mAbs, monoclonal antibodies; TLR, toll-like receptor. Created with BioRender.com.

Blood samples were collected between 8am and 12pm by nurses or physicians in tubes containing lithium heparinate. After receipt in the laboratory, one milliliter of whole blood was stimulated with immune agents that mimic the pathogen-associated molecular patterns that activate immune cells (R848 as TLR7/8 agonist and anti-CD3 as T-cell stimulant) in QuantiFERON-Monitor^®^ specific tubes (Qiagen^®^, Germany) within eight hours from blood collection. To measure IFN-γ levels produced by SARS-CoV-2-specific T cells, we used the QuantiFERON^®^ SARS-CoV-2 test (Qiagen^®^, Germany) in which one milliliter of whole blood was collected in tubes containing a mixture of SARS-CoV-2 peptides. Blood samples stimulated with SARS-CoV-2 specific and nonspecific immune agents were then incubated for 16 to 18 hours at 37°C and then centrifugated at 2000-3000 x g for 15 minutes to harvest the plasmas. Plasmas were then stored at –80°C until analysis and freeze-thaw cycles were minimized to preserve the quality of the samples. Plasma IFN-γ levels after stimulation were measured by enzyme-linked immunosorbent assay (ELISA). The procedure with nonspecific response is summarized in **Figure 1**. To note, when IFN-γ values obtained after a nonspecific response were above the limit of detection range (e.g., IFN-γ >1000 IU/mL), the values were arbitrarily scored as 1000 IU/mL. This functional test is simple to implement for both clinical and laboratory staff, making it applicable for routine use. In addition, for patients included *via* hospital consultations (CovImmune 1 study), we also measured plasma IL-6 by ELISA without cellular stimulation with custom-designed cartridges Ella (ProteinSimple™).

The proportions and numbers of CD4+ T cells, CD8+ T cells, NK cells and B cells, as well as their IFN-γ production with no stimulation or after nonspecific stimulation, were assessed in three healthy volunteers by flow cytometry. Eight milliliters of blood were collected: 2 mL with unstimulated cells, including 1 mL to measure IFN-γ by ELISA and 1 mL to identify IFN-γ-producing cells by flow cytometry, 2 mL with cells stimulated with TLR7/8 agonist, 2 mL with cells stimulated with anti-CD3, and 2 mL with cells stimulated with both TLR7/8 agonist and anti-CD3. After 4 hours of incubation, BD GolgiStopTM (0.66µL/mL) was added to the samples for flow cytometry, followed by an additional incubation time of 12 hours. Finally, cells were fixed and stained using fluorochrome-conjugated antibodies against surface molecules (CD45 BV786, CD5 PerCP-Cy5.5, CD2 FITC, CD7 BV711, CD3 BV510, CD8 PE, CD4 BV605, CD19 PE-Cy7, CD16 BV510, CD56-BV510, CD64 APC-H7) and intracellular molecules (CD3 APC, IFN-γ R718). All antibodies used are commercially available. Flow cytometry data were acquired on a BD FACSLyric™ and analyzed using BD FACSuite™ software. The gating strategy is depicted in **Supplementary Figure 1**.

### Statistical analyses

Data are presented as mean and standard deviation for quantitative variables with Gaussian distribution, as median and interquartile range [25th percentile; 75th percentile] for quantitative variables with non-Gaussian distribution, or as numbers and percentages for qualitative variables. The Shapiro-Wilk normality test was used to verify the distribution of data. Comparisons were performed using the unpaired two-sided Student’s t-test or Wilcoxon-Mann-Whitney U test according to data distribution for quantitative variables, and the Chi-square test for qualitative variables. The associations between specific and nonspecific IFN-γ responses, and between nonspecific IFN-γ response and plasma IL-6 values were compared using Spearman rank correlation coefficient. Multivariable logistic regression model was used to investigate independent factors that influence hospitalization. Receiver Operating Characteristic (ROC) curve was used to define an IFN-γ threshold below which patients would be considered at risk for hospitalization. Kaplan-Meier analysis was used to estimate the probability of hospitalization based on IFN-γ response. Statistical analyses were performed using GraphPad Prism 8 (GraphPad Software, Inc., San Diego, CA) for unadjusted analysis and Jamovi (version 1.8.4.0) for multivariable analysis. All comparisons were two-tailed, and the differences were considered significant when p value < 0.05.

## Results

### Interferon-γ production after nonspecific stimulation of innate and adaptive immune cells in healthy volunteers

**Figure 2:**
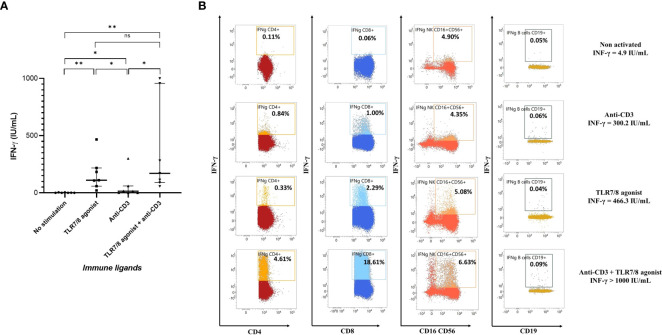
Evaluation of IFN-γ production after stimulation or not of circulating peripheral blood cells in healthy donors. **(A)** Plasma cytokine levels in seven healthy donors after *in vitro* nonspecific stimulation of immune cells by anti-CD3, or TLR7/8 agonist, or both anti-CD3 and TLR7/8 agonist, or no stimulation. IFN-γ production was mainly increased after TLR7/8 agonist stimulation and partially after anti-CD3 stimulation. **(B)** Representative plots of IFN-γ production by CD4^+^ T cells, CD8^+^ T cells, NK cells and B cells after stimulation or not with TL7/8 agonist and/or anti-CD3. As expected, after nonspecific stimulation CD4^+^ T, CD8^+^ T and NK cells produce more IFN-γ, but not B cells. The level of IFN-γ measured on the same individual and in the same conditions with no stimulation and after nonspecific stimulation with anti-CD3, TLR7/8 agonist, or both was 4.9, 300.2, 466.3 and >1000.0 IU/mL, respectively. *p<0.05; **p<0.01; ns, not significant. IFN, interferon; TLR, Toll-like receptor.

Immune cells of seven healthy donors were stimulated with immune ligands: TLR7/8 agonist as a stimulant of innate immune cells, anti-CD3 as T cells stimulant, both TLR7/8 agonist and anti-CD3, or no stimulation. As shown in **Figure 2A**, a very low level of IFN-γ was detected in plasma without cell stimulation. TLR7/8 agonist and anti-CD3 stimulations significantly increased IFN-γ production (p=0.0006 and p=0.03, respectively). Stimulation with the combination of a TLR7/8 agonist and an anti-CD3 significantly increased IFN-γ production (p=0.0006), but without a significant increase compared to the TLR7/8 agonist alone, probably due to a too small sample size.

The immune cells involved in IFN-γ production after nonspecific stimulation were innate immune cells, particularly NK cells, CD4^+^ T and CD8^+^ T cells (**Figure 2B**). Interestingly, we showed that the TLR7/8 agonist also activated CD8^+^ T cells (44, 45) and that the action of the two immune agents together seemed to be synergistic. There are probably large variations in the proportions and cell types activated after nonspecific stimulation from one individual to another (**Supplementary Figure 2**).

### Characteristics of the population and outcomes

**Figure 3:**
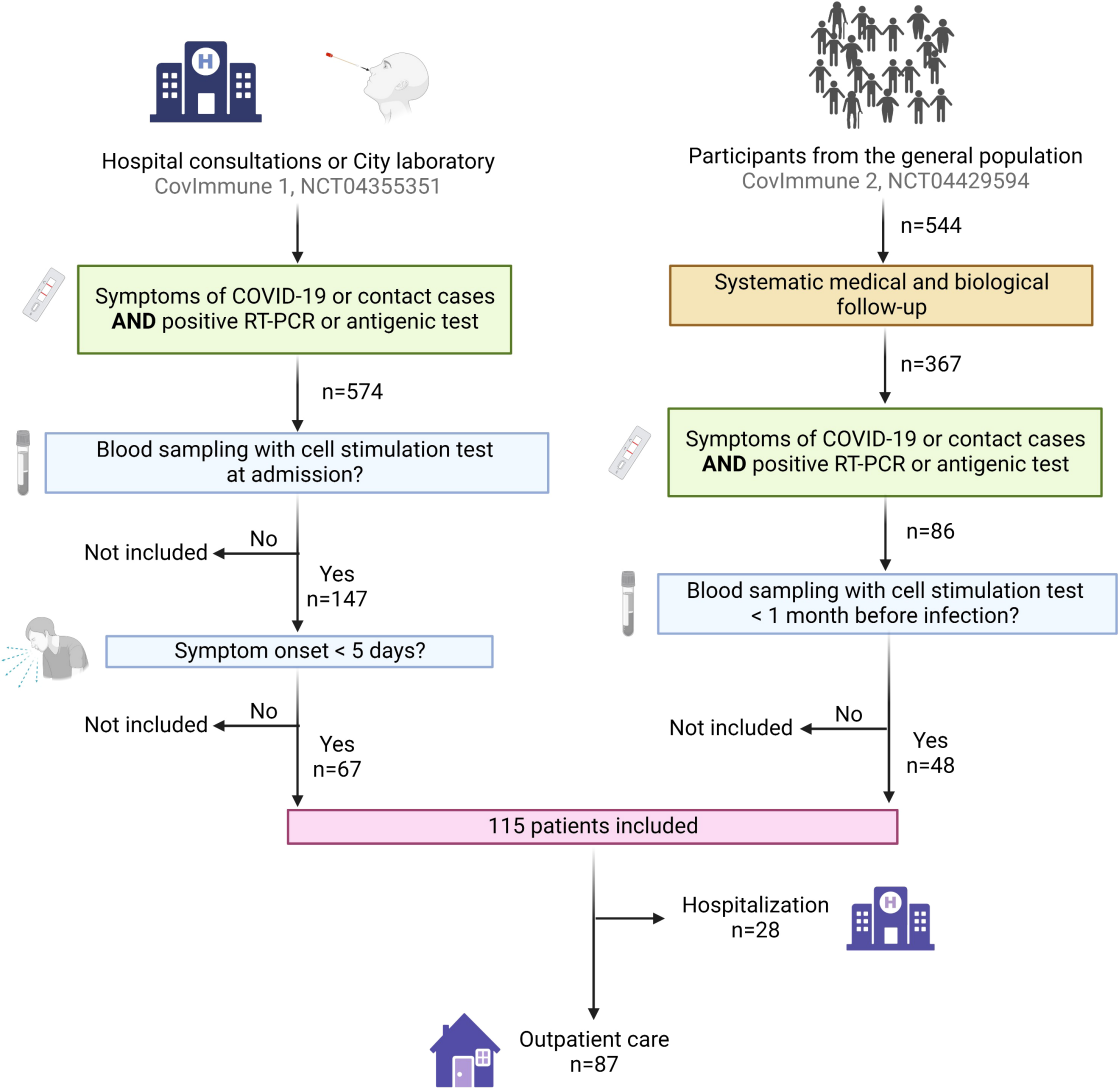
Flow chart showing participants enrollment. The participants were included from three cohorts: (i) patients recruited during a hospital consultation following COVID-19 symptoms, or as contact of a diagnosed COVID-19 case (CovImmune 1 study, NCT04355351); (ii) patients recruited by partner laboratories during a positive RT-PCR for SARS-CoV-2 (CovImmune 1 study, NCT04355351); (iii) participants monitored periodically since July 2020 as part of an epidemiological study in the context of COVID-19 (CovImmune 2 study, NCT04429594) and developing a SARS-CoV-2 infection. COVID-19 positive participants for whom we had a stimulated blood sample either a) within one month prior to infection or b) within five days after the first symptoms of the disease or after a close contact with a COVID-19 case, were enrolled in this study. COVID-19, coronavirus disease 2019; RT-PCR, reverse transcription-polymerase chain reaction. Created with BioRender.com.

We included 115 individuals infected with SARS-CoV-2 for whom we obtained a blood sample with nonspecific cell stimulation within one month prior to infection or within five days after the first symptoms during the period from March 2020 to January 2022 (**Figure 3**). The population’s characteristics are shown in **Table 1**. Sixty-six (57.4%) of the subjects were women, and the average age of the cohort was 53.9 (± 17.2) years. Sixty-three (54.8%) participants had at least one comorbidity. The most frequent comorbidities were hypertension (26.1%), immunosuppressive therapy (13.9%) and respiratory diseases (13.0%). Seventy-eight (67.8%) individuals were completely vaccinated. Common symptoms, including cough (47.0%), fever (39.1%) and dyspnea (26.1%), were reported by many individuals. Only five of them (4.3%) received antiviral treatment. Among the 115 individuals included in this study, twenty-eight (24.3%) subsequently progressed to severe pneumonia requiring hospitalization, 24 (20.9%) and 16 (13.9%) required oxygen therapy and corticosteroids, respectively, 11 (9.6%) were transferred to an intensive care unit, and five (4.3%) died. To note, among the 24 who required corticosteroids, 22 were hospitalized and two were outpatients.

**Table 1:** Population’s characteristics.

Population’s characteristics	Patients, n = 115
**Baseline characteristics**	
Sex	
Male	49 (42.6%)
Female	66 (57.4%)
Age, years	53.9 (± 17.2)
Comorbidities	
BMI (kg/m²)	24.9 [21.5-28.3]
Type 2 diabetes	11 (9.6%)
Cardiovascular diseases*	11 (9.6%)
Hypertension	30 (26.1%)
Respiratory diseases (COPD, asthma)	15 (13.0%)
Active cancer	9 (7.8%)
Immunosuppressive therapy	16 (13.9%)
SARS-CoV-2 vaccination**	78 (67.8%)
**Presentation at diagnosis**	
Symptoms	
Fever (>38·0°C)	45 (39.1%)
Cough	54 (47.0%)
Dyspnea	30 (26.1%)
Headache	27 (23.5%)
Myalgia	27 (23.5%)
Diarrhea	11 (9.6%)
Anosmia	19 (16.5%)
None	9 (7.8%)
Laboratory data	
Lymphocytes count (G/L)	1.1 [0.8-1.6]
Neutrophils count (G/L)	4.4 [2.9-6.3]
Hematocrit (L/L)	0.39 (± 0.64)
CRP (mg/L)	28.9 [6.4-106.6]
Serum creatinine (µmol/L)	77.0 [57.8-114.3]
IFN-γ levels after *in vitro* cell stimulation (IU/mL)	58.0 [15.0-234.0]
**Outcomes**	
Hospitalization	28 (24.3%)
Oxygen therapy	24 (20.9%)
Corticosteroids	16 (13.9%)
Intensive care unit	11 (9.6%)
Death	5 (4.3%)

The number (and percentage) are indicated for categorical variables, mean (and standard deviation) are shown for continuous variables with Gaussian distribution and median (and interquartile range) for continuous variable with non-Gaussian distribution. Lymphocyte and neutrophil counts, CRP and creatinine values were available for 59 patients. Hematocrit was available for 62 patients.

*heart failure or coronary artery disease.

**complete scheme (two or more injections of an mRNA vaccine, one or more injection of Janssen vaccine).

BMI, body mass index; COPD, chronic obstructive pulmonary disease; CRP, C-reactive protein; IFN-γ, interferon-gamma.

### Factors associated with the risk of hospitalization for COVID-19 patients

**Figure 4:**
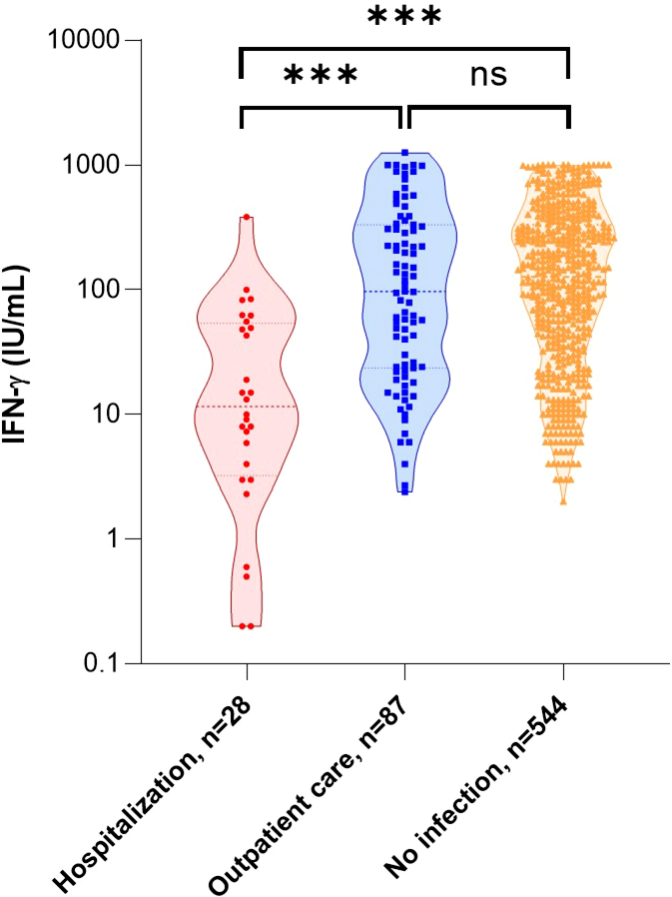
IFN-γ response and the risk of hospitalization after SARS-CoV-2 infection. Comparison of IFN-γ secretion by stimulated blood cells in relation to subsequent care for infection management and compared with no infection. Statistical significance of difference between groups was assessed using Mann-Whitney non-parametric test. ***p<0.001; ns, not significant. IFN, interferon.

As shown in previous studies (1–3, 46–49), unadjusted analysis of this cohort (**Table 2**) confirmed that the risk of hospitalization was significantly higher with increased age (p<0.001), male gender (p<0.001), and the existence of at least one comorbidity (p<0.001). No significant difference was found with body mass index (BMI) (p=0.083). As expected, the proportion of patients vaccinated was significantly lower in the group of patients requiring hospitalization (p<0.001). There was no difference in the lymphocytes count between the two groups (p=0.212), but a significant difference in the ability of immune cells to respond to a nonspecific stimulus, as measured by IFN-γ production, in those who were subsequently hospitalized (97.0 [interquartile range (IQR), 23.6-332.0] IU/mL vs 11.6 [IQR, 3.3-53.9] IU/mL, p<0.001) (**Figure 4**). Because we found a difference in hematocrit values between the two groups (p=0.022), we corrected for IFN-γ levels by performing a ratio with hematocrit values: again, patients who were subsequently hospitalized had a lower ability of immune cells to respond to a nonspecific stimulus (p<0.001). We then compared IFN-γ production between COVID-19 patients and 544 uninfected volunteers from the general population: we found no difference between outpatients and uninfected individuals (p=0.52) but a significant difference between hospitalized patients and uninfected individuals (p<0.001) (**Figure 4**). Of note, three patients in the outpatient group and ten uninfected individuals had IFN-γ above the limit of detection and the value was considered to be 1000 IU/mL. For information, the IFN-γ responses of volunteers from the general population, by age group and sex, are available as additional data (**Supplementary Table 1**).

**Table 2:** Clinical and biological presentation of COVID-19 patients according to clinical course.

	Outpatient care n = 87	Hospitalization n = 28	Univariable p value
**Demographic data**			
Age	50.1 (± 15.4)	65.5 (± 17.5)	<0.001
Sex (Male)	29 (33.3%)	20 (71.4%)	<0.001
BMI (kg/m²)	23.8 [21.2-27.6]	26.3 [24.7-29.4]	0.083
One or more comorbidities	38 (45.2%)	25 (89.3%)	<0.001
**Vaccine schedule**			
SARS-CoV-2 vaccination*	68 (59.1%)	10 (35.7%)	<0.001
**Biological data at presentation**			
Lymphocytes count (G/L)	1.2 [0.8-1.7]	1.0 [0.6-1.5]	0.212
Neutrophils count (G/L)	3.9 [2.9-5.0]	4.9 [3.2-7.4]	0.788
Hematocrit (L/L)	0.41 (± 0.52)	0.37 (± 0.69)	0.022
IFN-γ levels after *in vitro* cell stimulation (IU/mL)	97.0 [23.6-332.0]	11.6 [3.3-53.9]	<0.001
IFN-γ/hematocrit ratio (IU/mL)	270.5 [47.0-542.4]	36.3 [9.2-134.9]	<0.001

The number (and percentage) are indicated for categorical variables, mean (and standard deviation) are shown for continuous variables with Gaussian distribution and median (and interquartile range) for continuous variable with non-Gaussian distribution. Comparisons were performed using the unpaired two-sided Student’s t-test or Wilcoxon-Mann-Whitney U test according to data distribution for quantitative variables, and the Chi-square test for qualitative variables. Significant associations are highlighted.

*complete scheme (two or more injections of an mRNA vaccine, one or more injection of Janssen vaccine).

BMI, body mass index; IFN-γ, interferon-gamma. Bold value = p > 0.05.

Of the 115 patients, we performed IFN-γ assay after specific stimulation with SARS-CoV-2 peptides for 84 (73%) of them, of which ten were hospitalized and 74 were outpatients. Although there is a correlation between specific and nonspecific cellular responses (Spearman’s rho=0.246 [0.027; 0.443], p=0.024), we did not find an altered specific cellular response in patients who will subsequently be hospitalized (p=0.149). The data are shown in **Supplementary Figure 3**.

As confirmed by multivariable analysis, low stimulated IFN-γ levels were an independent predictor of hospitalization in COVID-19 patients (p=0.023), as were no vaccination (p<0.001) and being over the age of 65 (p=0.037). These results are detailed in **Table 3**.

**Table 3:** Factors independently associated with hospitalization for COVID-19.

	Adjusted odds ratio [95% CI]	Multivariable p value
**Demographic data**		
Age < 45 vs > 65 years	0.340 [0.043-2.694]	0.307
Age 45-65 vs > 65 years	0.129 [0.019-0.886]	0.037
Sex (Male)	3.424 [0.686-17.095]	0.134
One or more comorbidities	3.041 [0.567-16.302]	0.194
**Vaccine schedule**		
SARS-CoV-2 vaccination*	0.098 [0.025-0.390]	<0.001
**Biological data at presentation**		
IFN-γ ≤ 15 IU/mL	4.623 [1.231-17.361]	0.023

Multivariable logistic regression model was used to investigate independent factors that influence hospitalization. Significant associations are highlighted. Overall test model: p<0.001, R²=43.8%.

*complete scheme (two or more injections of an mRNA vaccine, one or more injection of Janssen vaccine).

CI, confidence interval; IFN-γ, interferon-gamma. Bold value = p > 0.05.

### A low IFN-γ response correlates with hospitalization in COVID-19 patients

**Figure 5:**
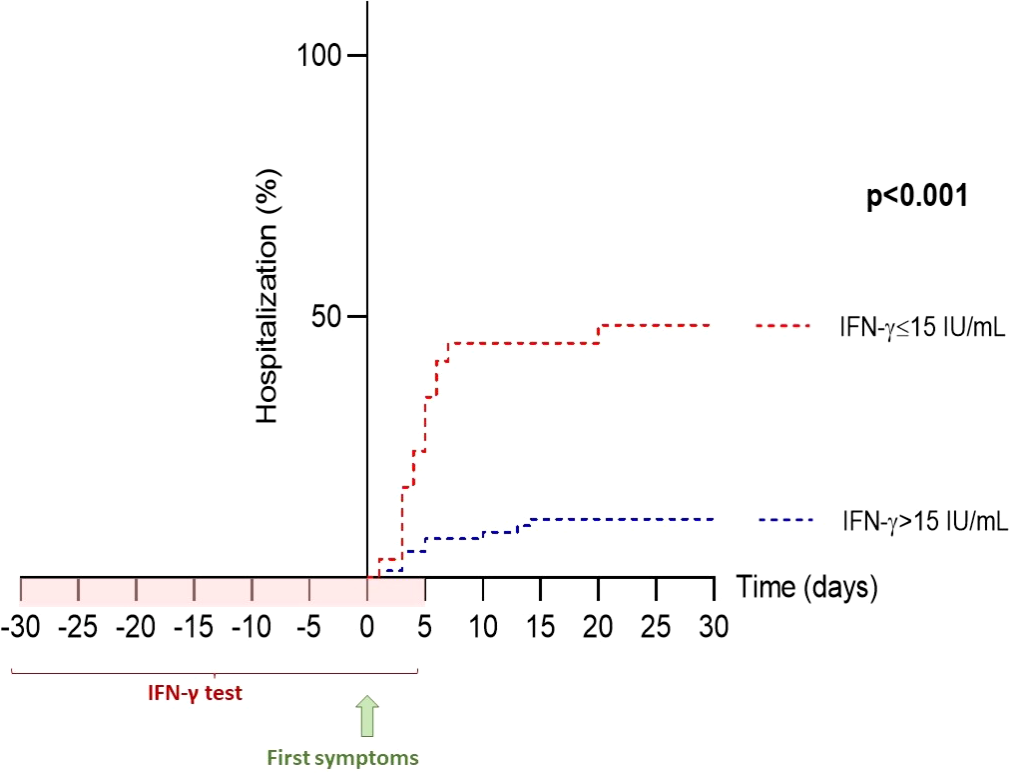
Hospitalization-free survival rate based on IFN-γ response. The IFN-γ threshold of 15 IU/mL, determined by ROC curve (sensitivity 67.9% and specificity 94.3%), was used for Kaplan-Meier analysis. Of the 32 patients with IFN-γ ≤15 IU/mL, 17 (53%) were subsequently hospitalized, whereas only 11 of 72 (15%) patients with IFN-γ >15 IU/mL were hospitalized. IFN, interferon; ROC, receiver operating characteristics.

**Figure 6:**
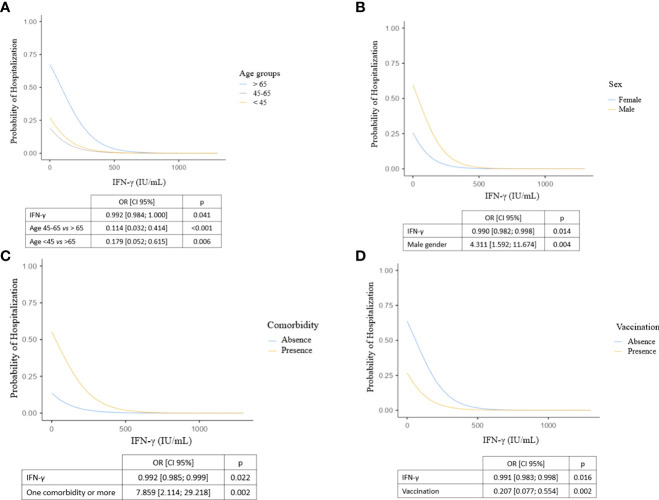
Probability of hospitalization according to IFN-γ levels, adjusted for **(A)** age class, **(B)** sex, **(C)** presence of at least one comorbidity and **(D)** vaccination. Logistic regression models including explanatory variables associated with hospitalization in multivariable analysis. We show here four representations of the model adjusted on the explanatory variables: each of them represents the impact of a factor and IFN-γ production on hospitalization after adjustment. CI, confidence interval; IFN, interferon; OR, odds ratio.

We then examined the utility of measuring IFN-γ response as a biomarker of hospitalization in the early phase of COVID-19 infection. We found a significant inverse correlation between IFN-γ response and hospitalization (odds ratio=0.990 [0.981; 0.996], p=0.007). Next, we evaluated the relevance of using IFN-γ response as a biomarker of hospitalization in COVID-19 patients using a receiver-operating characteristic (ROC) curve: the area under the ROC curve (AUC) was 87.9%, revealing a good performance of IFN-γ response in predicting hospitalization in COVID-19 patients. Using this ROC curve, we defined an IFN-γ cut-off value at 15 IU/mL to identify patients at risk of hospitalization (sensitivity: 67.9%, specificity: 94.3%, p<0.001). In univariable analysis, individuals with less than 15 IU/mL of IFN-γ after nonspecific stimulation were 6.57-times more likely to be subsequently hospitalized (odds ratio=6.57 [2.55; 16.95], p<0.001) (**Figure 5**). We then sought to clarify the predictive impact of the IFN-γ response by adjusting for variables independently associated with hospitalization in the multivariable model. We found that IFN-γ level was more predictive when the subject was older than 65 years, male, had at least one comorbidity and was not vaccinated (**Figure 6**). In other words, the risk of hospitalization in a young, healthy, vaccinated subject is low even if his or her IFN-γ is less than 15 IU/mL, whereas the risk is major in an individual with one or more associated factors who would have the same IFN-γ level. Thus, our data suggest that IFN-γ response, assessed by functional immunoassay, may be a tool for predicting the risk of hospitalization for early or subsequent SARS-CoV-2 infection.

### A low IFN-γ response correlates with a high level of IL-6 in plasma

For the 56 (48.7%) patients included *via* hospital consultations, we also had a plasma IL-6 assay without cell stimulation. Among them, 30 (53.6%) were outpatients and 26 (46.4%) were hospitalized. As expected, hospitalized patients exhibited more IL-6 levels than outpatients (40.70 [9.50; 71.60] vs 7.00 [0.99; 35.70] pg/mL respectively, p<0.001). Significant inverse correlation was found between IFN-γ response and plasma IL-6 (Spearman’s rho=-0.38 [-0.59; -0.13], p=0.003).

## Discussion

In this ancillary study of several prospective cohorts, we investigated whether an individual’s IFN-γ response, as assessed by a functional immunoassay, could predict the risk of hospitalization after SARS-CoV-2 infection. We found that participants with IFN-γ levels below 15 IU/mL were 6.57-times more likely to be hospitalized for COVID-19 than those with higher values. This risk was even higher if participants also had an associated factor such as age >65 years, male gender, presence of at least one comorbidity, or lack of vaccination. Probably due to a lack of statistical power, in the overall multivariable analysis, the relative risk of those under 45 years was not significantly associated with a lower risk of hospitalization than those over 65 years (**Table 3**), although it was in the logistic regression considering only the IFN-γ response and the age group (**Figure 6A**). Unlike other studies (4, 50), obesity was not found to be a risk factor for hospitalization, probably because few subjects were overweight in this cohort of individuals from southern France. In addition, we found a significant inverse correlation between IFN-γ response and plasma IL-6 in a subgroup of 56 patients, supporting data from other teams (24, 35, 41). This association supports the hypothesis that interferon deficiency may promote excess secretion of proinflammatory cytokines, probably due in part to the deficit in viral clearance. However, this result must be tempered by the fact that it is derived from a subgroup of patients and that the number of patients who have progressed to a severe form of COVID-19 requiring intensive care management is low. Further studies, including preclinical studies, are needed to demonstrate the impact of interferon variations on the inflammatory response in the context of SARS-CoV-2.

In this study, the SARS-CoV-2 specific cellular response was not associated with the risk of hospitalization. This result is not surprising given the study design. Indeed, in addition to the fact that the specific cellular tests were performed in only 84 patients, they were also performed very early after the infection, even before the contact with the infectious agent for almost half of the patients, including non-vaccinated patients for whom the specific response was necessarily non-existent without presaging their subsequent antiviral response. In our opinion, the evaluation of the specific cellular response is of particular interest in the evaluation of the post-vaccination immune response.

We believe that this simple to use functional measure of nonspecific cellular response could help clinicians identify patients who would benefit from early antiviral or IFN therapy, allowing for more personalized prescription. Moreover, the immune response must be maintained in a balanced manner and some recent data suggest that long COVID-19 may be due to an excessive IFN response (51). Thus, the choice of the molecule and the precise timing of its administration seem necessary both to induce viral clearance and prevent immunopathology. It could therefore be detrimental to prescribe IFN-based therapies in patients whose immunological balance is already dysregulated in favor of this pathway. Here again, this functional immunological test could help optimize management for a tailored prescription. Clinical trials could be considered: IFN or antiviral treatment if IFN-γ after cell stimulation <15 IU/mL in a patient with one or more associated factors but not within the indications of these therapies, to be compared to a standard-of-care arm.

The originality of this study is the analysis of the individual functional cellular response, at the early phase of the infection, and even before the infection for nearly half of the individuals included. If performed too late after the contamination, the results of the test would probably be modified by the ongoing anti-infectious response in various proportions: IFN-γ secretion increased at the peak of the antiviral response, decreased at the time of immune reconstitution and even more so in case of cell exhaustion, or even increased persistently in case of long COVID. As suggested by other teams, but without a functional approach (30, 38), a functional immunoassay performed early during the infection, or even before contamination, could predict the antiviral response against SARS-CoV-2, but also the response against other viruses or intracellular pathogens (52). This hypothesis is partly confirmed by our work but requires further studies.

Nevertheless, our study has several limitations. First, the inclusion period is long, extending from the beginning of the pandemic to a more recent period. It therefore covers infections with various variants of SARS-CoV-2 and pre- and post-vaccination periods. However, we are not studying the specific immune response to SARS-CoV-2 but the antiviral response in general, so the strain of virus should not influence the results. Also, vaccination was included in the multivariable model, which limits this bias. Secondly, the ability of immune cells to respond to stimulation also depends on an individual’s age, comorbidities, and infectious and immunological history (53). It is therefore difficult to disentangle these variables and establish IFN-γ standards. Finally, although the cohort size is reasonable given its prospective design and its biological assays with strict pre-analytical procedures, the sample size remains small, and the results need to be confirmed on a larger cohort.

Although many questions remain, early analysis of the IFN-γ response in a contact or recently infected subject could help the clinician choose the appropriate molecule for management and thus avoid severe forms of infection and hospitalization.

## Data availability statement

The raw data supporting the conclusions of this article will be made available by the authors, without undue reservation.

## Ethics statement

The studies involving human participants were reviewed and approved by Comités de Protection des Personnes Sud-Ouest et Outre-Mer 1 et 2, Agence Régionale de Santé - 10 Chemin du Raisin - 31050 TOULOUSE CEDEX 9. The patients/participants provided their written informed consent to participate in this study.

## Author contributions

BS-P designed the study; DG, VB, and MCr carried out experiments; MCr, BS-P, CF, KZ, JA, and CR-C collected data; MCr, KZ, JA, CR-C, MTi, and BS-P analyzed and interpreted the data; MCr and BS-P drafted and revised the manuscript. All authors contributed to the article and approved the submitted version.

## Funding

This research was supported by grants from the Agence Nationale de la Recherche (Flash-COVID ANR- 20-COVI-000) and Conseil Départemental des Alpes-Maritimes (CD06).

## Acknowledgments

We thank the medical and paramedical staff involved in the care of patients and all the participants.

## Conflict of interest

The authors declare that the research was conducted in the absence of any commercial or financial relationships that could be construed as a potential conflict of interest.

## Publisher’s note

All claims expressed in this article are solely those of the authors and do not necessarily represent those of their affiliated organizations, or those of the publisher, the editors and the reviewers. Any product that may be evaluated in this article, or claim that may be made by its manufacturer, is not guaranteed or endorsed by the publisher.
