# Supplementary figures and images for: Low baseline IFN-γ response could predict hospitalization in COVID-19 patients
Source: Front Immunol. 2022 Sep 26;13:953502. doi: 10.3389/fimmu.2022.953502 (PMC9548596; doi:10.3389/fimmu.2022.953502)

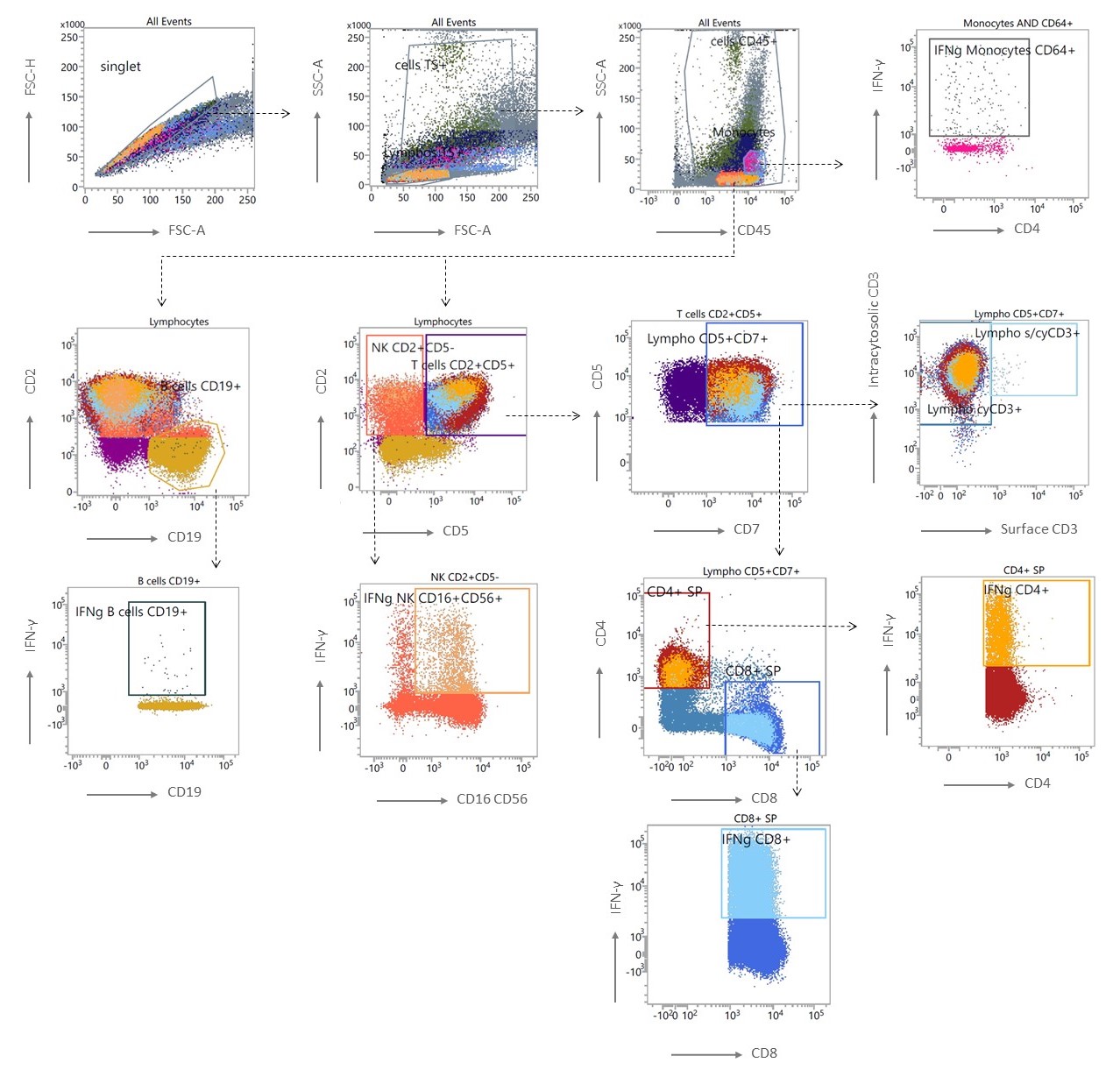

Supplement: Supplementary file 1 [file Image_1.jpeg]

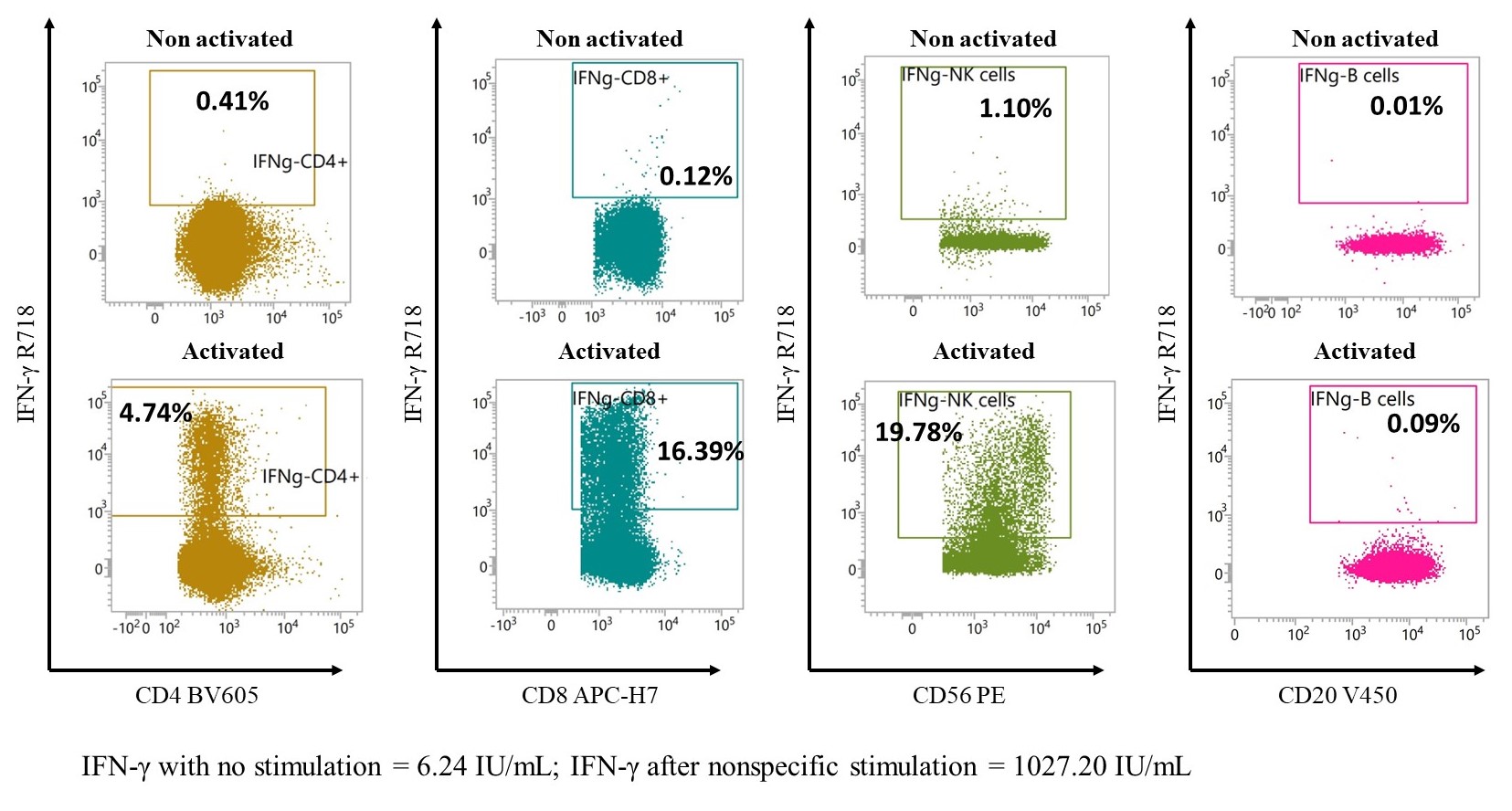

Supplement: Supplementary file 2 [file Image_2.jpeg]

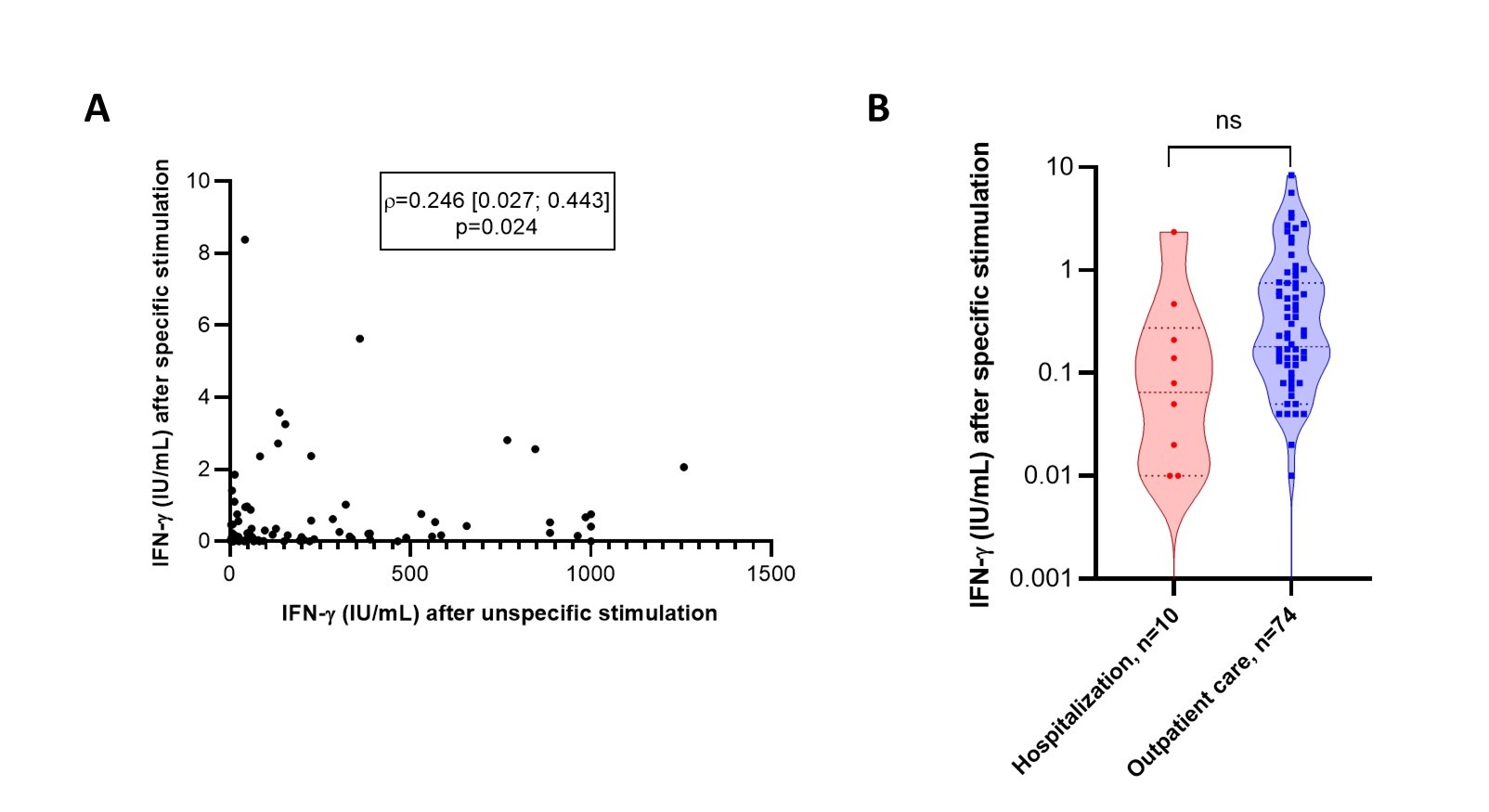

Supplement: Supplementary file 3 [file Image_3.jpeg]
