# Supplementary material for: Low baseline IFN-γ response could predict hospitalization in COVID-19 patients
Source: Front Immunol. 2022 Sep 26;13:953502. doi: 10.3389/fimmu.2022.953502 (PMC9548596; doi:10.3389/fimmu.2022.953502)
Supplement: Supplementary file 4 [file DataSheet_1.docx]

**Supplementary figure legends**

**Supplementary figure 1: Gating strategy for identification of IFN-γ producing cells in healthy donors after *in vitro* nonspecific stimulation of immune cells.** Immune cells were stimulated with anti-CD3, TLR7/8 agonist, both anti-CD3 and TLR7/8 agonist, or with no stimulation. Here we used plots with both anti-CD3 and TLR7/8 agonist to show gating strategy. Due to the internalization of CD3 after anti-CD3 stimulation, dual intracytoplasmic and surface CD3 staining were performed. Monocytes were gated for size, structure and CD64 expression. To note, the three healthy donors analyzed by flow cytometry had low levels of monocytes, despite normal blood count values, probably due to the experimental conditions. Thus, although it is known that monocytes secrete IFN-γ, the quantity secreted was negligible after *in vitro* nonspecific stimulation given the experimental conditions.

**Supplementary figure 2: Plots in a second individual of IFN-γ production by CD4^+^ T cells, CD8^+^ T cells, NK cells and B cells after stimulation or not with TL7/8 agonist and anti-CD3.** As expected, after nonspecific stimulation CD4^+^ T, CD8^+^ T and NK cells produce more IFN-γ, but not B cells. The level of IFN-γ measured on the same individual and in the same conditions with no stimulation and after nonspecific stimulation was 6.24 and 1027.20 IU/mL, respectively.

**Supplementary figure 3: Specific cellular response and risk of hospitalization in SARS-CoV-2 infection.**

**(A) Correlation between specific and unspecific IFN-γ responses.** The association between specific and unspecific responses were compared using Spearman rank correlation coefficient. **(B) Comparison of IFN-γ secretion after peripheral blood cell-specific stimulation with SARS-CoV-2 peptides in relation to subsequent care.** Statistical significance of difference between groups was assessed using Mann-Whitney non-parametric test.

IFN-γ, interferon-gamma; ns, not significant.

**Supplementary table 1: IFN-γ levels after *in vitro* cell stimulation in a sample of uninfected individuals from the general population**

|  | IFN-γ (IU/mL) |
| --- | --- |
| All individuals, n=544 | 126.5 [35.0-330.8] |
| Age |  |
| < 45 | 149.0 [47.0-393.0] |
| 45-65 | 114.0 [32.0-291.5] |
| > 65 | 152.0 [17.0-244.5] |
| Sex |  |
| Female gender | 122.0 [32.5-315.5] |
| Male gender | 143.0 [57.0-407.0] |

IFN-γ levels after *in vitro* cellular stimulation are presented as median and interquartile range [25e percentile-75e percentile] by age group and sex in a sample of uninfected individuals from the general population.

IFN-γ, interferon-gamma.
